# Ribosome profiling-guided depletion of an mRNA increases cell growth rate and protein secretion

**DOI:** 10.1038/srep40388

**Published:** 2017-01-16

**Authors:** Thomas Beuchert Kallehauge, Shangzhong Li, Lasse Ebdrup Pedersen, Tae Kwang Ha, Daniel Ley, Mikael Rørdam Andersen, Helene Faustrup Kildegaard, Gyun Min Lee, Nathan E. Lewis

**Affiliations:** 1 Novo Nordisk Foundation Center for Biosustainability, Technical University of Denmark, 2800 Kgs. Lyngby, Denmark; 2 Department of Bioengineering, University of California, San Diego, La Jolla, CA 92093, United States; 3 Novo Nordisk Foundation Center for Biosustainability at the University of California, San Diego, School of Medicine, La Jolla, CA 92093, United States; 4 Department of Systems Biology, Technical University of Denmark, 2800 Kgs. Lyngby, Denmark; 5 Department of Biological Sciences, KAIST, Daejeon 305-701, Korea; 6 Department of Pediatrics, University of California, San Diego, School of Medicine, La Jolla, CA 92093, United States

## Abstract

Recombinant protein production coopts the host cell machinery to provide high protein yields of industrial enzymes or biotherapeutics. However, since protein translation is energetically expensive and tightly controlled, it is unclear if highly expressed recombinant genes are translated as efficiently as host genes. Furthermore, it is unclear how the high expression impacts global translation. Here, we present the first genome-wide view of protein translation in an IgG-producing CHO cell line, measured with ribosome profiling. Through this we found that our recombinant mRNAs were translated as efficiently as the host cell transcriptome, and sequestered up to 15% of the total ribosome occupancy. During cell culture, changes in recombinant mRNA translation were consistent with changes in transcription, demonstrating that transcript levels influence specific productivity. Using this information, we identified the unnecessary resistance marker NeoR to be a highly transcribed and translated gene. Through siRNA knock-down of NeoR, we improved the production- and growth capacity of the host cell. Thus, ribosomal profiling provides valuable insights into translation in CHO cells and can guide efforts to enhance protein production.

The primary determinants of protein expression are (1) expression levels of the host and recombinant genes, (2) translation efficacy, and (3) proper folding and post-translational processing of relevant proteins. We can now begin to understand the molecular basis of protein expression thanks to recent efforts to sequence the genomes of cell lines utilized for recombinant expression^1^,^2^,^3^. Specifically, RNA sequencing (RNA-Seq)^2^,^4^,^5^,^6^,^7^,^8^,^9^,^10^,^11^ and proteomics^12^,^13^ have provided detailed insights into transcription and post-translational processes; however, the translation of endogenous and recombinant mRNA has remained poorly characterized. This is of particular concern since RNA-Seq analyses neglect the fact that translation control of the mRNA is an essential and highly regulated step in determining levels of an expressed protein^14^.

To begin untangling the complex regulation of translation and how plastic translation is upon introduction of an exogenous mRNA, ribosome profiling (Ribo-Seq) has emerged and precisely measures translation globally *in vivo*, thereby offering a method to exactly determine which mRNAs are actually being translated^15^,^16^, and at which rates. At the center of this technique lies the observation that ribosomes protect ~30 nucleotides of an mRNA from nuclease digestion^16^,^17^. Purification followed by deep sequencing of these ribosome-protected fragments (RPFs), allows precise mapping of the position of a ribosome on an mRNA at a given time point. The density of RPFs on a given coding sequence often correlates with the rate of protein synthesis^16^.

Investigations into the translational landscape of mammalian expression hosts have been sparse. A combination of polysome profiling and microarray analyses has previously been used to study the effect of mTOR pathway manipulation on cellular growth, global translation, and recombinant protein expression^18^,^19^,^20^. These studies provided valuable insights into the translational state of the cell, but they are limited by the sensitivity of the techniques and do not provide information regarding the exact ribosome positioning on a single transcript level or how a recombinant mRNA integrates into- and affects the translated endogenous mRNA pool.

Here, we present the first comprehensive genome-wide view of the translational activity of a mammalian expression host during recombinant expression by the application of ribosome profiling. Since Chinese hamster ovary (CHO) cells have been a primary host for producing complex recombinant proteins, thanks to their capability to often correctly fold and post-translationally modify human proteins^21^,^22^, we used these cells as our model system. The distribution of translational power was analyzed in the context of the recombinant mRNAs and the endogenous mRNA pool. In an antibody-producing cell line, the recombinant mRNAs were found to be the most abundant transcripts and also sequestered a substantial amount of translating ribosomes (up to 15%). The recombinant mRNAs were translated as efficiently as the endogenous mRNAs, and changes in translation and transcription of the recombinant mRNAs were directly reflected in changes in specific productivity. Interestingly, improvements in bioprocess quality attributes were achieved by depleting the highly expressed and translated unnecessary NeoR mRNA by siRNA-mediated knock-down. This resulted in improved cellular growth, which was accompanied by an 18% increase in antibody titers. This study is the first to carefully map the translatome of the CHO cell to the nucleotide level and to demonstrate how recombinant expression affects the cell on the translational level.

## Results

### Ribosomal occupancy was measured in IgG-producing CHO cells

The CS13-1.0 CHO cell line was previously developed to overexpress a chimeric antibody against S surface antigen of hepatitis B virus^23^, and the DHFR system was employed to amplify the antibody. Thus, the cell line harbors approximately 90 gene copies per cell of the heavy chain encoding gene and approximately 280 gene copies per cell of the light chain encoding gene^23^.

**Figure 1:**
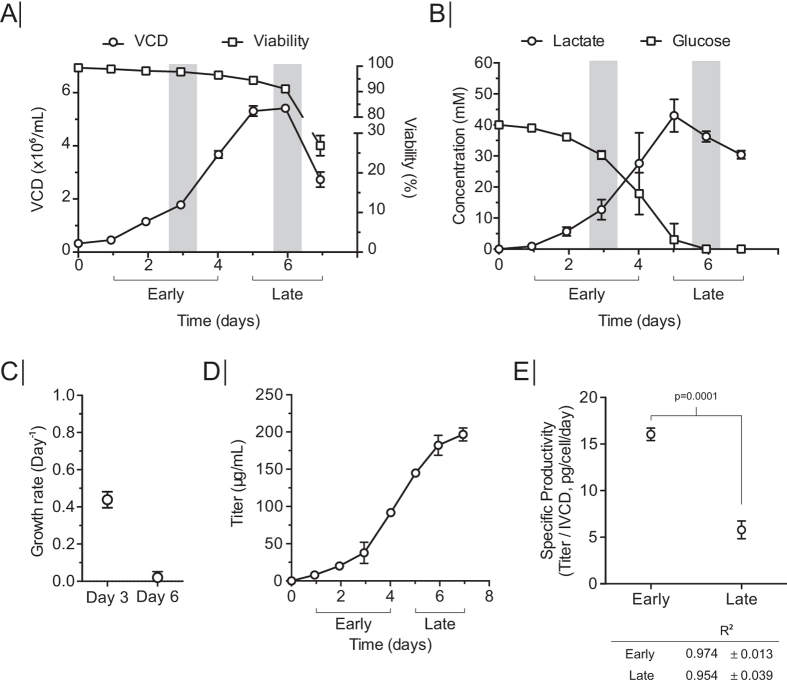
Batch culture for ribosome profiling. (**A**) Viable cell density (VCD) and viability during batch culture. (**B**) Lactate and glucose concentration in growth medium during batch culture. (**C**) Specific growth rate, (**D**) antibody titer, and (**E**) specific productivity (*q*_*p*_) in early and late growth phase. Linear regression from Qp calculation for each growth phase presented below graph. Error bars in all plots depict standard deviation (*n* = 3).

To emulate a production relevant growth setting, the CS13-1.0 CHO cell line was grown in a batch culture bioreactor system, and the cells were sampled at early and late growth phase for ribosome profiling to study the translatome during IgG-production. Cells were grown for seven days until reaching a maximum viable cell density of ~5.5 × 10^6^ cells/mL (Fig. 1A). The cultivation was divided into two phases: an early growth phase from day one to day four, and a late growth phase from day five to day seven. Samples were taken for ribosome profiling (Ribo-Seq) and RNA-Seq within the two growth phases, on day three and day six respectively. These time points were chosen as they represented two different metabolic conditions and growth rates: (1) exponential growth on glucose and (2) as cells are shifting from growing on glucose to growing on lactate in stationary phase (Fig. 1B,C). Antibody titers were measured daily, using biolayer interferometry, reaching ~196 μg/mL (Fig. 1D), which was consistent with previously published data^24^. Specific productivity (*q*_*p*_) at day three was calculated to be 16 picogram/cell/day (pcd) and 5.5 pcd at day six, which also reflected a significant metabolic difference between day three and day six (Fig. 1E).

### Ribosome profiling shows translation activity of recombinant and host proteins with nucleotide resolution

**Figure 2:**
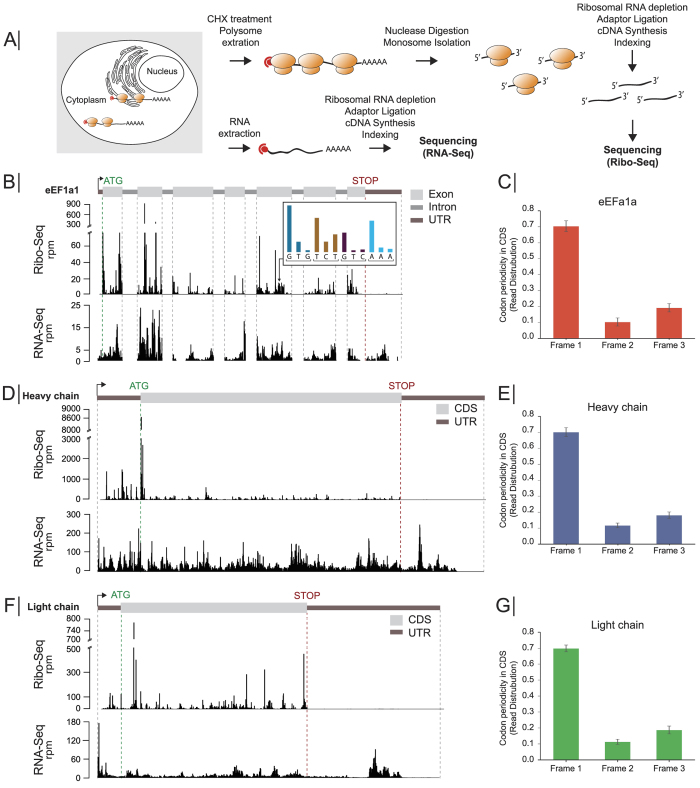
Ribosome profiling of CHO cells. (**A**) Schematic representation of ribosome profiling workflow in which Ribo-Seq and RNA-Seq data were simultaneously generated. (**B**) Read depth along the genes from Ribo-Seq (top track) and RNA-Seq (bottom track) of eEF1a1 on day 3. Insert depicts the read distribution along ORF. Brackets indicate translated ORF codon. (**C,E,G**) Codon periodicity seen from Ribo-Seq data in the open reading frame of eEF1a1, heavy chain, and light chain. Ribo-Seq and RNA-Seq read distribution on day 3 are shown for the (**D**) heavy chain, and (**F**) light chain. RPM: transcripts per million, CDS: coding sequences.

Samples harvested on days three and six were subjected to ribosome profiling and RNA-Seq in biological triplicates (Fig. 2A). Briefly, monosomes were generated from cyclohexamide (CHX)-stalled polysomes by nuclease treatment. The ~30 nucleotide RPF fragments were then sequenced to generate the Ribo-Seq data set. In parallel, total RNA isolated from the same cells was prepared for RNA-Seq. Both RNA-Seq and Ribo-Seq data were aligned to the CHO reference genome^3^.

The ribosome profiling data differed from classical RNA sequencing, as shown for the endogenous eEF1a1 (Fig. 2B). Both the Ribo-Seq and RNA-Seq data mapped along the exons, with few reads in the introns. However, unlike the RNA-Seq data, the Ribo-Seq showed almost no coverage in the 3′UTR region, since upon arrival at the stop codon, the ribosome dissociates from the mRNA. As ribosome footprints define the sequence being translated, a periodic pattern is frequently observed along the mRNA in the Ribo-Seq (Fig. 2B, insert). This three-nucleotide pattern of ribosome occupancy, or codon periodicity can be seen along the ORF (Fig. 2C), with ~70% of the RFPs starting at the first nucleotide of the codon. This reflects mRNA translocation in the ribosome by codon as translation occurs^16^,^25^. Similar features were seen for the recombinant antibody heavy- and light chain (Fig. 2D,F). The codon periodicity of the heavy- and light chain were not only consistent with that of eEF1a1 (Fig. 2E,G), but also with the codon periodicity across all translated endogenous proteins (Fig. S2). Additionally, the ribosomal stalling frequency along the CDS of the heavy- and light chain mRNA did not differ from that of the endogenous mRNA pool (Fig. S3).

**Figure 3:**
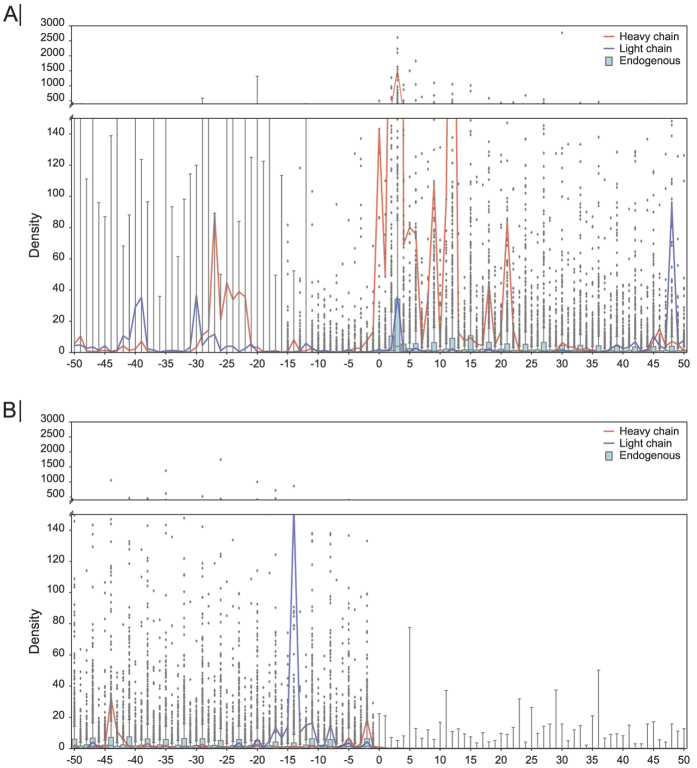
Ribosomal densities around translation start- and stop sites on day three. (**A**) Ribosomal densities around the translational start site. Red- and blue lines represent the heavy- and light chain mRNA, respectively. The boxplot at each position represents the normalized ribosomal density of all endogenous mRNAs. (**B**) Normalized ribosomal densities around the translational stop site. Ribosomal densities for each mRNA were normalized by the median density of the given mRNA.

To get a measure of how ribosomes distribute around the translational start- and stop codon of the heavy- and light chain mRNA compared to endogenous mRNA, ribosomal occupation around the start and stop codon for day three was mapped (Fig. 3A,B). Interestingly, both the heavy- and light chain had significantly more reads in the 5′UTR compared to the endogenous mRNAs (Fig. 3A, Supplementary statistical analysis to Fig. 3). No reads were observed in the 3′UTR of the heavy- and light chain mRNA, which also was the case for the endogenous mRNAs (Fig. 3B). Collectively, these data indicate that the recombinant IgG mRNAs have a ribosome distribution comparable to the endogenous mRNAs, with an exception of having higher ribosomal occupancy in the 5′UTR of the recombinant mRNAs.

### A global view of translation during recombinant expression

**Figure 4:**
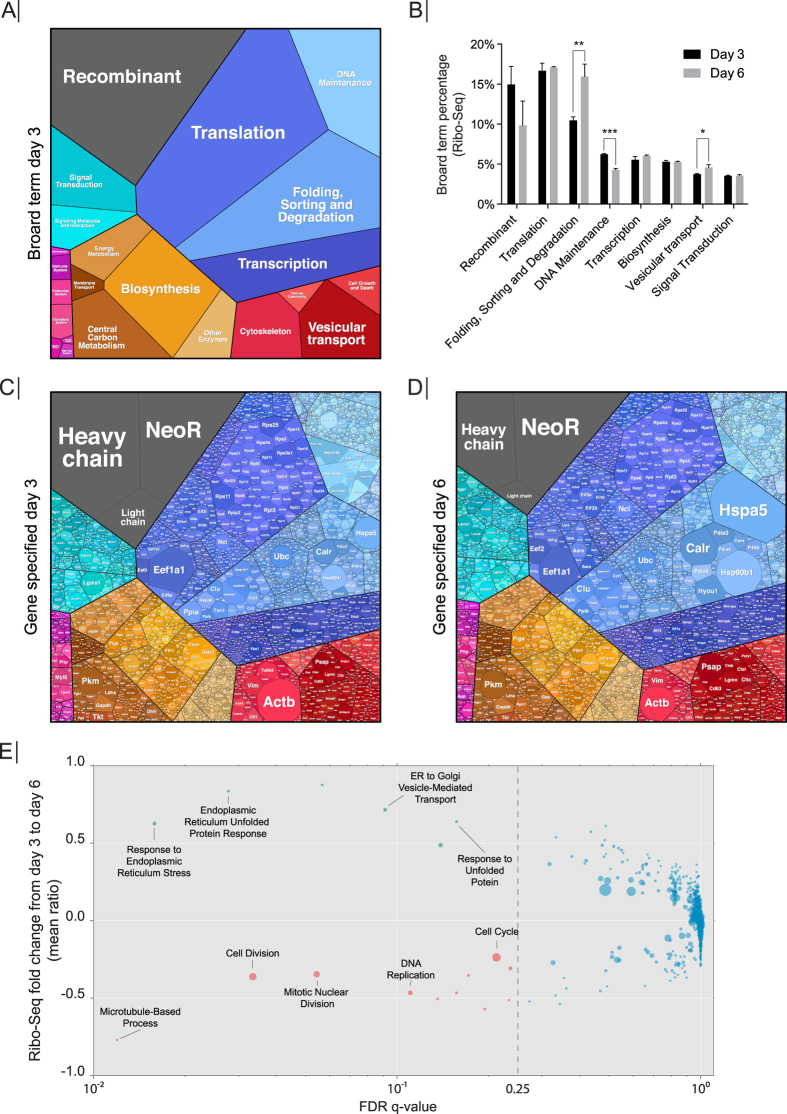
Distribution of translational power across cellular processes. (**A**) Ribo-Seq reads were grouped into their corresponding cellular processes (data shown are from day 3). The area of each polygon represents the absolute ribosomal occupancy (not normalized to transcript length). (**B**) The quantity of Ribo-Seq reads of major cellular processes changed from day 3 to day 6. Error bars depict standard deviation (*n* = 3). *P*-values were determined by Student’s *t*-test (two tailed). *P < 0.02, **P < 0.005, ***P < 0.0002. (**C,D**) The absolute ribosomal occupancy (not normalized by transcript length) is shown for all individual genes, based on the Ribo-Seq data from (**C**) day 3 and (**D**) day 6. (**E**) Between days 3 and 6, ribosomal occupancy significantly changed for many genes. The genes were divided into GO terms (based on Gene Set Enrichment Analysis) and plotted to highlight the gene families that showed significant changes in their translation. The False Discovery Rate (FDR)^48^ was plotted on the x-axis and the mean of the fold change for the each GO term on the y-axis. Highlighted are GO terms with a significant FDR below 0.25 and a mean ratio that was respectively positive (upregulated, green) or negative (downregulated, red). The size of each point is relative to the number of gene members in the GO term.

A goal in bioprocessing is to maximize recombinant protein yields. However, the maximum reported cell-specific productivities (*q*_*p*_) still pale in comparison to professional secretory cells *in vivo*, such as pancreatic or salivary cells. Thus, we analyzed how the translational power of the CHO cell was distributed across all host and recombinant genes, and the cellular processes to which they contribute. We binned the reads from the Ribo-Seq into categories corresponding to the cellular process and visualized the genes and their processes using Proteomaps (Fig. 4; see Materials and Methods for details)^26^,^27^. Proteomaps visually groups genes into cellular processes, and therefore provides a global view of total ribosomal occupancy (not scaled by transcript length, rpm). The recombinant mRNAs were among the most abundant in the Ribo-Seq data occupying 14.96% of Ribo-Seq reads on day three, but dropped to 9.8% on day six (Supplementary Table S5) (Fig. 4A,B). Transcripts associated with the Translation category dominated the ribosomal occupancy of the endogenous mRNAs, steadily sequestering 16.67% on day three and 17.09% on day six (Supplementary Table S5) (Fig. 4A,B).

Among the individual recombinant mRNAs, the heavy chain mRNA sequestered 7.73% of the Ribo-Seq reads on day three, but only 3.86% on day six (Fig. 4C,D) (Supplementary Table S6). The light chain mRNA displayed the same trend by occupying 1.60% on day three, but only 0.58% on day six. Interestingly, the antibiotic selection marker, initially used to generate the stable cell line (Kim *et al*.^23^), was found to sequester a substantial amount Ribo-Seq reads (Fig. 4C,D). The NeoR mRNA remained relatively stable by sequestering 5.63% and 5.41% on day three and day six, respectively (Supplementary Table S6). Interestingly, the DHFR gene, which was also part of the plasmids used to generate the CS13-1.0 cell line^23^, only accounted for 0.0002% and 0.0001% of the RNA-Seq reads on day 3 and 6, respectively (Supplementary Table S6, see “Comment to Fig. 4” in the Supplementary materials).

The major rearrangement in distribution of translational power between day three and day six happened between the Recombinant category, which dropped ~5% from 14.96% to 9.98% (*P* = 0.08), and Folding, Sorting and Degradation category, which displayed a ~5% increase from 10.50% to 16.0% (*P* = 0.004) (Fig. 4A,B) (Supplementary Table S5). Within the Folding, Sorting and Degradation category, Hspa5 (BiP) and Hsp90b1 were the dominant mRNA species, sequestering 2.81% and 1.2% of the Ribo-Seq reads, respectively (Fig. 4D) (Supplementary Table S6). The concurrent drop and increase between these two categories suggests that the translational power is redirected from recombinant expression to the proteins involved in the secretory pathway. Based on these observations, fold changes in the Ribo-Seq between day three and day six were calculated (Supplementary Table S7), and a Gene Set Enrichment Analysis (GSEA) was performed to identify biological processes that significantly changed their translational status (Fig. 4E, Supplementary Table S8). GO-terms with decreased ribosomal occupancy included processes involved in cellular growth and cell cycle, which is consistent with the cells transitioning to stationary phase at day six (Fig. 1A). Of the six significantly upregulated GO-terms, four were associated with ER stress. The upregulation of ER-stress markers could be ascribed to the depletion of glucose on day six (Fig. 1B), which has previously been described to induce ER stress^28^. This increased translation of ER stress markers is further supported by increased protein expression of the ER stress marker BiP in the late growth phase (Fig. S4).

When considering the general distribution in the RNA-Seq data, the recombinant mRNAs constitute 20.18% of all mRNA in the cell on day three and 12.38% on day six (Supplementary Table S5), while they account for 14.96% to 9.98% of the Ribo-Seq reads, respectively. This difference between the Ribo-Seq and RNA-Seq distribution highlights the importance including Ribo-Seq data for an exact analysis of the gene expression in CHO cells^6^, and further presents a unique view into how the cell distributes its translation power.

### Recombinant mRNA is translated as efficiently as the endogenous mRNA pool

The sequence of a protein-coding gene and mRNA includes a wealth of information beyond the amino acid code. Regulatory elements, RNA binding protein motifs, and sequences that influence RNA structure can all impact mRNA translation^29^. Our lack of understanding of these sequence elements has limited our ability to explicitly consider them in transgene design and codon optimization. Therefore, it is unclear if transgenes are translated as efficiently as the host cell proteome, or if unknown regulatory elements negatively impact translation.

**Figure 5:**
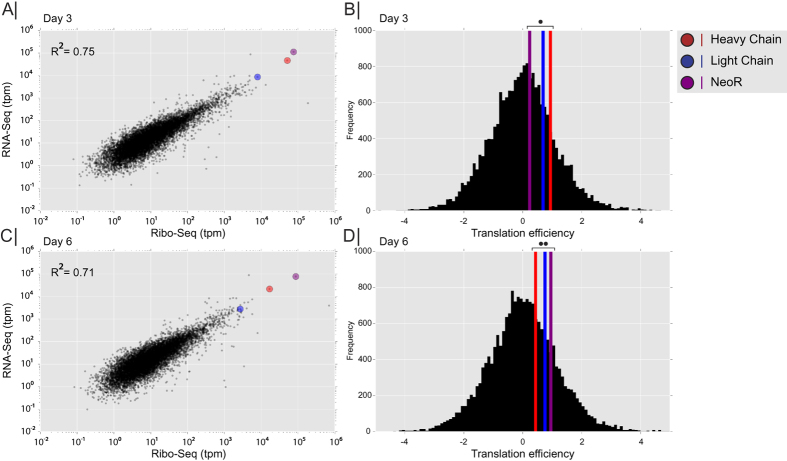
Ribosome footprint correlates with RNA abundance. (**A,C**) Scatter plot of Ribosome footprint density (Ribo-Seq) plotted against mRNA abundance (RNA-Seq) on day 3 and 6. Recombinant mRNA highlighted in colored circles (red: heavy chain, blue: light chain, purple: NeoR). (**B,D**) Histogram of translational efficiency (the ratio of ribosome footprint density to mRNA density) on day 3 and day 6. The colored vertical lines indicate the translation efficiency of the recombinant genes (red: heavy chain, blue: light chain, purple: NeoR). Two tailed t-test p-value was calculated based on the assumption that recombinant and endogenous genes have similar translational efficiency. Day three (•) *P*-value for Heavy chain = 0.41, Light chain = 0.55, and NeoR = 0.83. Day six (••) *P*-value for Heavy chain = 0.73, Light chain = 0.54, and NeoR = 0.44.

To address the question of translatability, we computed the translation efficiency^16^, which is defined by the ratio of ribosomal occupancy compared to transcript levels (ratio of ribosome footprint to mRNA fragments, both scaled by length, tpm) for each host and recombinant gene in our cell lines (Fig. 5). By plotting the ribosome footprint density (Ribo-Seq, tpm) against mRNA abundance (RNA-Seq, tpm), a linear relationship was observed between these two parameters thereby demonstrating that translation efficiency is constant for endogenous genes showing both low and high expression (Fig. 5A,C) (Supplementary Table S7). Genes showing translation efficiency (i.e., a ratio of Ribo-Seq to RNA-Seq) that was either significantly higher or lower than expected, were enriched in genes involved in RNA processing and translation, as assessed using GSEA (Supplementary Tables S9–10).

We subsequently examined if the translation efficiency for recombinant proteins was comparable to average host cell mRNA translation, or if their translation efficiency deviated significantly from the host mRNA translation. Statistical analysis showed that their standard deviation was within less than 1 of the endogenous translation efficiency values (Fig. 5B,D). These results suggest that ribosomal occupancy on the recombinant genes expressed here were not strongly limited by factors beyond transcript level.

### Changes in translatability and transcript levels are reflected in specific productivity

**Figure 6:**
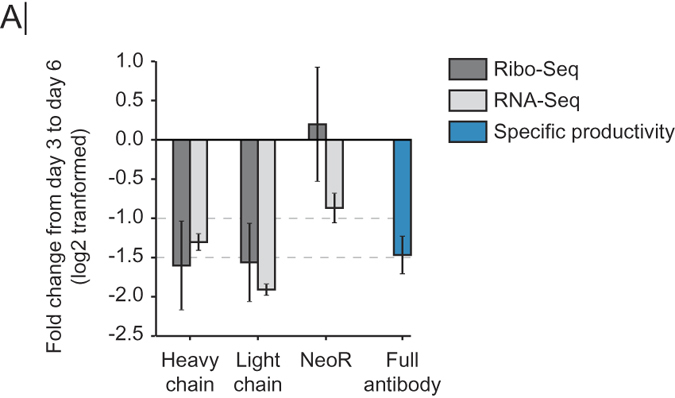
Specific productivity linked to transcription. (**A**) Fold change in RNA-Seq, Ribo-Seq and specific productivity. Values are the mean of 3 replicates and error bars are standard deviation.

Protein secretion can vary drastically between growth phases and the metabolic shifts involved^30^. However, it is unclear if this is a result from changes in transcription or translation of the recombinant gene. During the metabolic shift (Fig. 1B), many transcripts and RPFs significantly changed in abundance. The recombinant gene transcript levels and ribosomal density (normalized to gene length) showed an approximate 3 fold downregulation (Fig. 6A, Supplementary Table S7). Since the changes in translation follow the change in transcription, it suggests that the transcription levels from the recombinant genes in this study do not saturate the translational capacity. As further support, the specific productivity likewise drops 3 fold, pointing at transcription levels having the largest influence on specific productivity in this study.

### Depletion of highly expressed NeoR mRNA improves growth and antibody titers

**Figure 7:**
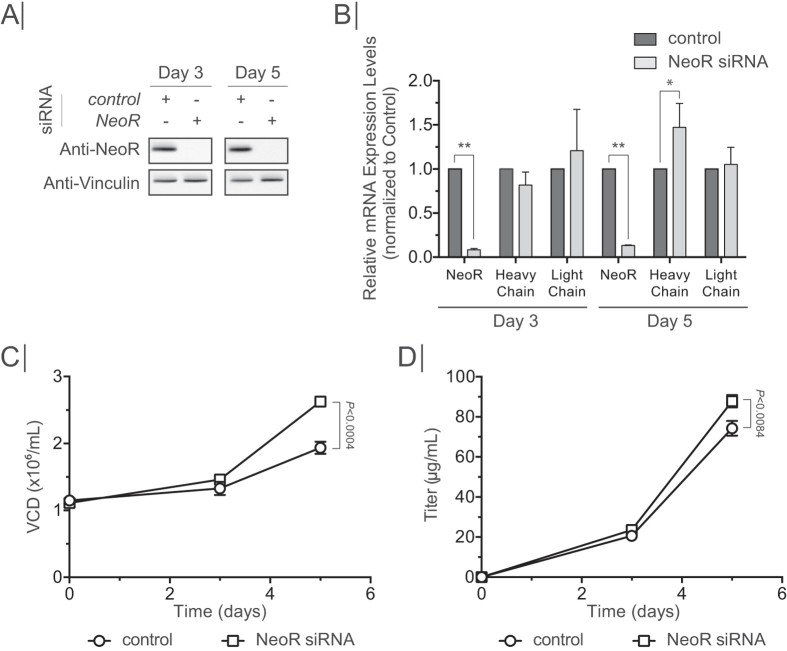
Knock-down of NeoR. (**A**) Western blot showing knock-down efficiency on protein level in cell treated with either Control siRNA or NeoR siRNA from day 3 and day 5 post transfection. Vinculin presented as loading control. (**B**) RT-qPCR showing knock-down efficiency on mRNA level. Values were normalized to control siRNA. *P < 0.04, **P < 0.0001. (**C**) Viable cell density of cultures treated with control or NeoR siRNA. (**D**) Antibody productivity of cultures treated with control or NeoR siRNA. Error bars depict standard deviation (*n* = 3). *P*-values were determined by Student’s *t*-test (two tailed).

In addition to the recombinant protein of interest, host cells spend a substantial amount of resources on producing other proteins, such as host cell proteins^13^,^31^,^32^. Indeed, our cell lines demonstrated that ~90% of the ribosomal occupancy is spent on proteins other than the desired IgG (Fig. 4). Thus, it is possible that the specific productivity could be substantially improved by eliminating genes that compete with protein production resources. To test this, we depleted the NeoR mRNA, as it was the dominant mRNA species in both the transcriptome and translatome (Fig. 4). Since cells were cultivated in the absence of Neomycin, any negative effect of NeoR mRNA depletion on cellular physiology was not expected by the NeoR depletion. CS13-1.0 cells were transiently transfected with siRNA against NeoR mRNA and a negative control. Knock-down efficiency was measured on day three and day five post transfection. NeoR protein was depleted on days three and five post transfection with no effects observed from the negative control siRNA (Fig. 7A). NeoR mRNA levels were reduced by 92% and 87% on day three and day five, when compared to negative control, respectively (Fig. 7B). No significant changes, other than a 47% upregulation of heavy chain mRNA levels on day five, were observed in the heavy and light chain mRNA transcript levels (Fig. 7B). Intriguingly, the viable cell density was increased by 35% upon NeoR mRNA knock down, which was accompanied by an 18% increase in product titers (Fig. 7C,D). These results show that a depletion of a highly abundant mRNA encoding a non-essential protein can improve growth and thus product titer, indicating that it is possible to free up translational capacity.

## Discussion

The recent advances in genomics and RNA sequencing technology now enable in-depth analyses of mammalian cells producing recombinant proteins. Here, we mapped out the distribution of translational power in a recombinant cell line through the use of ribosome profiling. This new technique has proven to be a powerful tool for precise mapping of unannotated ORFs^16^,^33^,^34^, identifying local translational slowdown from non-optimal codons, to improved signal recognition particle (SRP) recognition of the nascent polypeptide chain^35^, and measuring localized translation at the ER and the mitochondria^36^,^37^. Here we extended the field of application and employed ribosomal profiling to study the expression and translation of recombinant proteins in CHO cells.

Out of ~14000 mRNA species sequenced, the RNA-Seq and Ribo-Seq data showed that the three recombinant mRNAs made up 20% of the total mRNA content and sequestered up to 15% of all ribosomes engaged in translation. The recombinant mRNAs were efficiently integrated into the endogenous mRNA pool as their translation efficiency was similar to the endogenous mRNAs. The translational capacity was not biased for or against the recombinant proteins, as the ribosome density and transcript levels display a linear relationship for the host and recombinant proteins. This observation was supported by the findings that the specific productivity and transcript levels of the antibody showed a proportional relationship. Since degradation of the recombinant proteins and residence time in the secretory pathway are important factors in relation to the specific productivity^38^, future work could aim to explore how these factors work together to affect recombinant expression.

Interestingly, even though the recombinant mRNAs were the dominant mRNAs in the cell, other endogenous mRNAs displayed a higher degree of translation efficiency. It remains to be shown, however, whether translation becomes a limiting factor or if biases against the recombinant protein arise when cells are grown under industrial conditions with higher protein production rates.

A recent study showed that it was possible to elevate the steady-state recombinant mRNA levels from ~16% to ~30% by treating with enhancers of specific productivity^6^. The increase in recombinant mRNA concentration was accompanied by a proportional increase in specific productivity. The amount of recombinant mRNA present in the non-enhancer treated samples was consistent with our results, together with the proportional correlation of transcript level and specific productivity. Interestingly, in the Fomina-Yadlin study, the specific productivity of untreated cells reached ~22 pcd, whereas in our study it only reached 16 pcd, even though the steady-state recombinant mRNA levels were the same. It would be interesting to analyze the translation efficiency of the recombinant mRNAs in the enhancer-treated cells and compare it to our data to evaluate whether it is the translatability of the individual recombinant mRNA, or if it is the translational capacity of the other cell line, which can ascribe for the differences.

The production of unnecessary proteins has previously been found to have a negative impact on cell growth^32^. In the CS13-1.0 cell line, the NeoR, a leftover remnant from the original cell line construction^23^, was found to be the dominant mRNA species in the cell, and sequestered a significant portion of translating ribosomes. With NeoR being a non-essential gene at this point, depletion of the NeoR mRNA resulted in an increase in cellular growth, which was accompanied, by an increase in product titer. This indicates that it is possible to modulate the translation by removing non-essential mRNAs, thus freeing up translational capacity to be distributed throughout the translatome. Since the siRNA depleted NeoR mRNA without, most likely, reducing transcription, the effects observed could be ascribed to an increase in ribosome availability. Thus, depletion of a major non-essential mRNA may free up translational capacity, which could then be distributed to other beneficial cellular processes, e.g. growth and recombinant protein production. With the NeoR mRNA depletion being carried out under suboptimal growth conditions, a stable insertion of an shRNA would alleviate the stress of transfection. In turn, this would allow cultivation under optimal conditions, which would likely improve the results obtained. Furthermore, our findings highlight the need of careful consideration when choosing a cell line construction strategy to avoid highly expressed non-essential genes, which will end up sequestering a substantial amount of translational capacity.

Ribosome profiling has provided a better understanding of how a mammalian expression host utilizes and distributes its translational power both on endogenous- and exogenous mRNAs and how the transgene integrates into the steady-state mRNA pool. To expand our knowledge even further of how recombinant expression affects the endogenous mRNA population, it would be of great benefit to conduct further ribosome profiling studies comparing parental cell lines in parallel with derivative recombinant cell lines, generated by targeted insertion of a single expression cassette of different recombinant proteins. This setup would provide a controlled environment to evaluate the translational effects of expressing different recombinant proteins. In general, ribosome profiling of the wild type CHO cells would provide essential information about 5′UTR features influencing translation efficiency, and motifs that affect translational pausing. Such valuable information could aid in constructing future cell lines for recombinant expression. Recently, ribosome profiling has been used to highlight the importance of correct codon choice in a heterologous transcript in yeast^39^. Similar experiments performed in CHO cells would be very beneficial to further improve recombinant expression since the ribosome profiling of the CHO cell helps to link the transcriptome with the proteome, thus generating a comprehensive overview of the central dogma in CHO cells.

## Materials and Methods

### Cell culture and bioreactor cultivations

CS13-1.0 CHO cells expressing chimeric IgG against S surface antigen of hepatitis B virus^23^ were maintained in PowerCHO-2 serum free media (Lonza #12-771Q) supplied with 4 mM glutamine (Lonza #17-605 F) in vented Erlenmeyer shake flasks (Corning, NY) in a shaking incubator operated at 37 °C, 5% CO_2_ and 120 rpm. Additionally, cells were supplemented with 0.2% anti-clumping agent (Life Technologies #0010057AE) and, during maintenance, 1 μM Methotrexate hydrate (Sigma #M8407-100MG). Due to high degree of clumping, cells were passed through a 40 μm Corning cell strainer (Sigma #CLS431750-50EA) at each passage.

CS13-1.0 cells were cultivated in 1 L (SR0700) bioreactors (Eppendorf DASGIP, Jülich, Germany) with a working volume of 600 mL. Temperature was maintained at 37 °C with an agitation rate of 200 rpm using one three-way segmented impellers. Dissolved oxygen was maintained at 50% of air saturation using air, N_2_, O_2_ and CO_2_ operated at a constant flow rate of 0.6 L/h. Culture pH was maintained at 7.15 with a dead-band of 0.15 using intermittent CO_2_ addition to the gas mix and 2 M sodium carbonate. Culture pH and pO_2_ was measured on-line. Cell number and viability were measured using a hemocytometer with tryphan blue staining (Sigma-Aldrich). Batch cultures were seeded with 0.3 × 10^6^ cells/mL in triplicates and samples were drawn on a daily basis. On day 3 and day 6 samples were taken for ribosome profiling. The culture was terminated after 170 hours.

### RNA purification and RT-qPCR

RNA was extracted from a minimum of 1 × 10^6^ cells using Trizol (Life Technologies #15596-026) following manufacturer’s protocol. For qPCR analysis, cDNA was made from 500ng TURBO-DNase (Life Technologies #AM1907) treated RNA, using the qScript Flex cDNA kit (Quanta Bioscience # 95049-100) with random priming. qPCR was run in an Mx3005 P (Agilent Technologies) using Brilliant III Ultra-Fast SYBR^®^ Green master mix (Agilent Technologies # 600882). Oligos for qPCR are listed in Supplementary table S1. Fold changes was calculated using the ∆∆CT method. GAPDH was used as reference gene in all calculations.

### Retrieval of the heavy- and light chain gene sequence

The recombinant heavy- and light chain genes were sequenced to enable mapping of RNA-Seq and Ribo-Seq read mapping. See Supplementary Experimental Procedures for full protocol. cDNA was made from TURBO-DNase, Trizol extracted RNA from CS13-0.02 cells^23^ using qScript Flex cDNA kit with random priming. A forward primer, annealing just downstream of the CMV transcription start site, and a reverse primer annealing just upstream of the BgH polyadenylation signal, was used to amplify the genes. PCR products were cloned into a Zero Blunt TOPO vector (Life Technologies #450245) and sequenced. Sequence for heavy- and light chain including CMV promoter and BgH terminator sequence available in Supplementary Table S2.

### Ribosome profiling

Ribosome profiling was performed using the ARTseq/TruSeq Ribo Profile kit (Illumina #RPHMR12126) according to manufacturer’s protocol with few alterations. 25 × 10^6^ cells were harvested from bioreactor and immediately treated with cycloheximide (CHX, final concentration of 100 μg/mL) and incubated at RT for 3 minutes. Cells were pelleted at 1000 × g and washed with ice-cold PBS containing 100 μg/mL CHX. Cells were then handled as described in the manufacturer’s protocol. RPFs were purified on 10% Novex^®^ TBE-Urea gels (65 minutes at 200 V) (Life Technologies #EC6875BOX). The generated cDNA was gel-purified on 10% Novex^®^ TBE-Urea gels. The final PCR amplified libraries were all gel purified on 10% Novex^®^ TBE-Urea gel to remove excessive amounts of adaptor-dimer. Library quality was verified using the Agilent High Sensitivity DNA Assay (Agilent Technologies #5067-4626). Libraries were sequenced with TruSeq chemistry on a HiSeq 2500 platform (Illumina) in a 50 bp single read run. Raw data will be available at the Gene Expression Omnibus (GSE79512) upon publication.

### Sequence alignment

Sequence data for both RNA-Seq and Ribo-Seq were quality controlled using FastQC^40^ and then Trimmomatic^41^ was used to trim adapter sequences and low quality bases from the reads. Sequence alignment was accomplished using Bowtie2^42^ and Tophat2^43^. Specifically, rRNA sequences were eliminated by aligning reads to CHO-K1 rRNA sequences using Bowtie2. Reads that did not align to rRNA were then aligned to CHO-K1 reference genome using Tophat2. If Tophat2 reports multiple alignments with the same best quality score, we randomly chose one hit by setting the parameter -g as 1.

### Ribo-Seq quantification

Ribo-Seq quantities represent the number of reads that start at a given base pair. If the translated mRNA is in the sense strand of the sequenced genome, the 5′ end of aligned reads represents the 5′ end of ribosome. For mRNAs that are encoded in the antisense direction, the 3′ end of an aligned read represents the 5′ end of the ribosome. Thus, to quantify ribosomal occupancy, read counts at each base pair of a gene were based on the 5′ end of aligned reads for sense mRNA and based on the 3′ end of aligned reads for antisense mRNAs. Ribo-Seq reads varied in length (Supplementary figure S1) and each length represents a different offset for the ribosomal A site. To recalibrate the distance between the 5′ end and the ribosomal A site, we analyzed the sequence coverage around the translation start site and the translation stop site for every alignment length. The offset for each alignment length is provided in Supplementary table S3. After recalibration of the A sites, we assessed the ribosome occupancy periodicity by calculating the percent coverage at each reading frame. This was done by summing the coverage at all bases of each frame, divided by total coverage of the whole coding sequence (CDS).

The ribosomal occupancy surrounding the translation start and stop sites was analyzed. To this end we identified the transcript with the longest coding sequence for each gene, and then quantified the total read count at each coding position of that transcript to represent the corresponding abundance. For each representative transcript, we calculated the median coverage of its codons, after excluding the first 15 and last 10 codons. Transcripts with a median ribosomal occupancy of their codons that is greater than 0 in at least one replicate sample were used for the analysis of the translational start and stop sites. For each gene, the coverage at each nucleotide of the interested regions was normalized by 1/3 of their corresponded proteins’ median codon coverage. Finally, median values across all normalized coverage at each position were plotted. Furthermore, all claims in our study are based on gene-level quantities, or higher-level groups of genes. Therefore, the expected level of error is expected to be very low^44^ (much lower than the 5% changes seen in our data for the cited categories).

### Proteomaps

Ribosomal occupancy was calculated in the unit of reads per million reads (rpm). Since Proteomaps^26^,^27^ does not have a database for CHO genes, we mapped all CHO-K1 genes to their mouse homologs using three methods to maximize coverage of overlapping CHO and mouse genes. Specifically, we first conducted a two-way BLAST to map all transcript sequences between both CHO and mouse using BLAST + 2.2.29^45^. Second, we applied Inparanoid 4.1^46^ to identify homologs between CHO and mouse based on protein sequences that were extracted from Refseq 68. Third, we identified genes with exactly the same full gene names in the CHO-K1 and mouse annotations and treated them as homologs. The final merged ID mapping between CHO and mouse is listed in Supplementary Table S4. If a CHO gene exhibits high homology to multiple mouse Entrez gene IDs, the gene abundance was evenly allocated to all mapped mouse IDs. The abundance of reads for each gene was then submitted to Proteomaps. While Proteomaps does not include annotation for all mouse genes, nor do all CHO genes have mouse homologs, ~80% of total ribosomal occupancy, representing 11680 CHO genes, is accounted for by Proteomaps. Thus, the dominant cellular processes are quantified and show the global distribution translation in the cells.

### Gene Set Enrichment Analysis (GSEA)

GSEA was performed using the Broad Institute GSEA software^47^,^48^. GO terms for CHO were obtained using GOCHO^13^ and terms under the parent term ‘Biological Process’ were extracted and converted into a GMT file according to GSEA manual. A ranked list of genes was made using the expression values obtained from Ribo-Seq quantification. These data were run through the GSEA pre-ranked protocol. GSEA-pre-rank analysis was processed to detect significant GO terms whose genes that demonstrated an abnormal Ribo-Seq/RNA-Seq ratio (i.e., showing more or less ribosome occupancy then expected) (Supplementary Tables S9 and S10).

Explanation to why NeoR and not the DHFR mRNA is the dominating selection marker present in the ribosome profiling data is presented in the supplementary material (“Comment to Fig. 4”).

### siRNA knock down

siRNA duplexes against NeoR were designed using the Custom RNAi Design Tool at the IDT web site (Supplementary table S1). Silencer^®^ Negative Control No. 1 siRNA (Life Technologies #AM4611) was used as negative control. siRNAs were transfected into CHO cells with FreeStyle^TM^ MAX reagent (Invitrogen) according to the manufacturer’s protocol to a final concentration of 10 mM. Briefly, one day before transfection, cells were seeded at 0.5 × 10^6^ cells/mL of seeding density in PowerCHO-2 medium (Lonza) supplemented with 4 mM glutamine and without anti-clumping agent (Life Technologies). For transient transfection of siRNAs, cells were centrifuged and resuspended in 10 mL FreeStyle^TM^ CHO expression medium (Invitrogen) containing 8 mM glutamine at 1 × 10^6^ cells/mL of seeding density. FreeStyle^TM^ MAX reagent and siRNA duplexes for each target gene were mixed in the Opti-MEM (Invitrogen) as follows: 40 μL of siRNA stock (10 μM) was gently diluted in 160 μL Opti-MEM. 30 μL FreeStyle MAX reagent was gently diluted in 170 μL Opti-MEM and incubated at RT for 5 min. After 5 min, the siRNA mix and FreeStyle MAX reagent mix was combined to a total volume of 400 μL and incubated for 20 min at RT. Transfection mix was then added to the 10 mL cell culture. 4 hr after transfection, the cells were centrifuged and resuspended in PowerCHO-2 medium at 0.3 × 10^6^ cells/mL of seeding density into 125 mL Erlenmeyer flasks containing 20 mL of culture medium. Transfected cells were harvested at days 3 and 5 for further analysis.

### Immunoblotting

Cell pellets for protein extraction were washed once in PBS, and then lysed in Lysis Buffer (10 mM Tris pH 8.0, 137 mM NaCl, 7.5 mM MgCl_2_, 1% NP-40, 1x complete mini EDTA free (Roche #04693159001)). Proteins were separated on 4–12% NuPAGE Novex Bis-Tris gels (Life Technologies #NP0321BOX) with PageRuler Plus Prestained Protein Ladder (Thermo Scientific #26619). Protein samples were prepped for electrophoresis by boiling samples in 1xNuPAGE^®^ LDS Sample Buffer (Life Technologies #NP0007) and 1xNuPAGE^®^ Sample Reducing Agent (Life Technologies #NP0004). Protein was transferred to a nitrocellulose membrane using the iBlot^®^2 Transfer Stack nitrocellulose mini kit (Life Technologies #IB23002) using manufacturer’s protocol. Protein transfer was verified by staining with Ponceau S (Sigma #P7170). Blots were blocked with 5% skimmed milk powder in PBS + 0.1% Tween-20 (Sigma #P1379). All antibody incubation was carried out in 5% skimmed milk powder in PBS + 0.1% Tween-20. Antibodies applied: Vinculin (1:1000, Sigma #V9131), Neomycin Phosphotransferase 2 antibody [4B4D1] (1:1000, AbCam #ab60018), and BiP (1:1000, Cell Signaling Technology #C50B12).

### Octet quantification

To quantify chimeric antibody against S surface antigen of hepatitis B virus, biolayer interferometry was performed using an Octet RED96 (Pall Corporation, Menlo Park, CA). ProA biosensors (Fortebio 18-5013) were hydrated in PBS and preconditioned in 10 mM glycine pH 1.7. A calibration curve was prepared using human IgG at 250, 125, 62.5, 31.3, 15.6, 7.8 and 3.9 μg/ml. Antibody supernatants were collected after centrifugation and association was performed for 120 s with a shaking speed of 200 rpm at 25 °C. Octet System Data Analysis 7.1 software was used to calculate binding rates and absolute antibody concentrations.

## Additional Information

**How to cite this article**: Kallehauge, T. B. *et al*. Ribosome profiling-guided depletion of an mRNA increases cell growth rate and protein secretion. *Sci. Rep.* **7**, 40388; doi: 10.1038/srep40388 (2017).

**Publisher's note:** Springer Nature remains neutral with regard to jurisdictional claims in published maps and institutional affiliations.

## Supplementary Material

Supplementary Information

Supplementary Table 4

Supplementary Table 5

Supplementary Table 6

Supplementary Table 7

Supplementary Table 8

Supplementary Table 9

Supplementary Table 10
