# Supplementary material for: Ribosome profiling-guided depletion of an mRNA increases cell growth rate and protein secretion
Source: Sci Rep. 2017 Jan 16;7:40388. doi: 10.1038/srep40388 (PMC5238448; doi:10.1038/srep40388)
Supplement: Supplementary Information [file srep40388-s1.pdf]

Supplementary material

Ribosome profiling-guided depletion of an mRNA increases cell growth rate and protein secretion

Thomas Beuchert Kallehauge, Shangzhong Li, Lasse Ebdrup Pedersen, Tae Kwang Ha, Daniel Ley, Mikael Rørdam Andersen, Helene Faustrup Kildegaard, Gyun Min Lee‡, Nathan E Lewis‡

Supplementary Figure S1 Read length distribution of ribosome profiling
Supplementary Figure S2 Global codon periodicity
Supplementary Figure S3 Global stall site frequency
Supplementary Figure S4 Increased BiP expression at late growth phase
Comment to Figure 4
Supplementary statistical analysis to Fig. 3

Supplementary Table S1: Oligo and siRNAs
Supplementary Table S2: Heavy- and light chain mRNA sequence
Supplementary Table S3: Ribosome profiling alignment offset length
***Supplementary Table S4:** Merged ID mapping between CHO and mouse
***Supplementary Table S5:** Broad term percentage division of Ribo-Seq and RNA-Seq
***Supplementary Table S6:** Gene specific percentage division Ribo-Seq and RNA-Seq division
***Supplementary Table S7:** Mean ratio, Fold change and Translatability data
***Supplementary Table S8:** GSEA of fold changes from day three to day six
***Supplementary Table S9:** Translation Efficiency grouped by GSEA_Day3
***Supplementary Table S10:** Translation Efficiency grouped by GSEA_Day6

Reference

* See accompanying spreadsheets

Figure S1

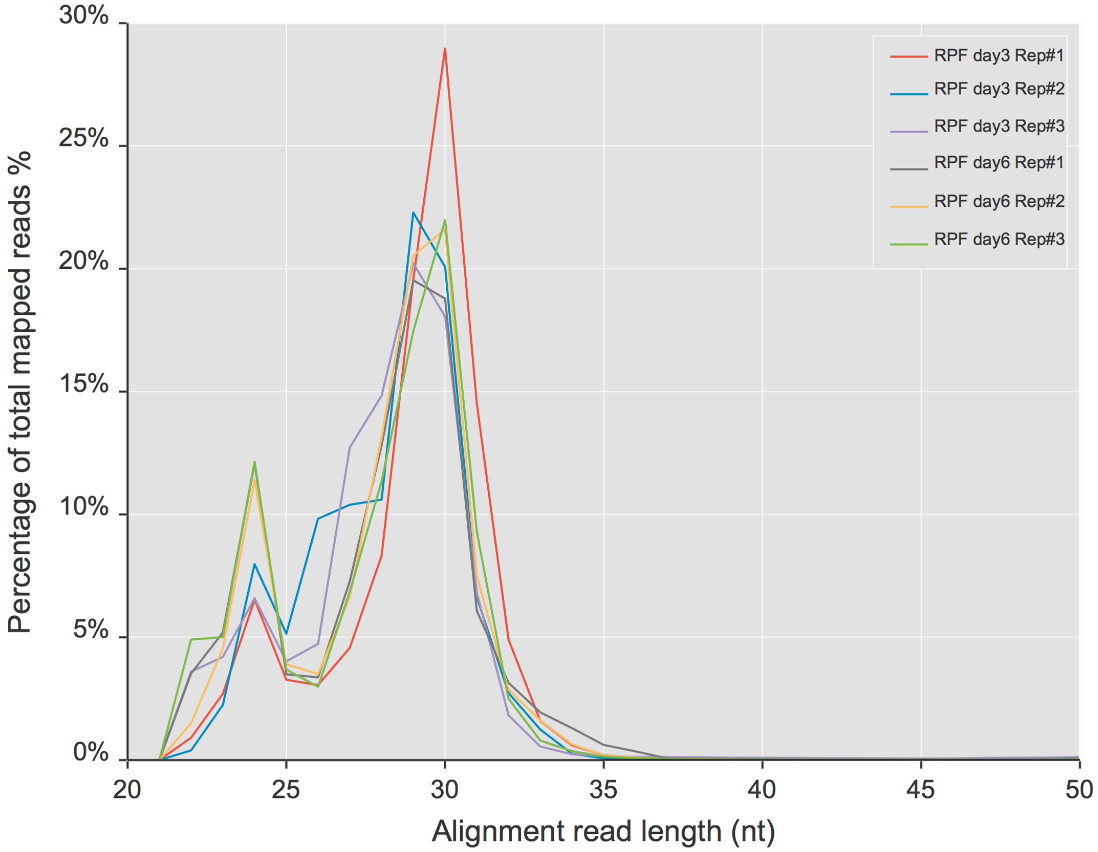

Supplementary Figure S1 Read length distribution of ribosome profiling. All three replicates from day 3 and day 6 presented. RPF: Ribosomal profiling fragments.

Figure S2

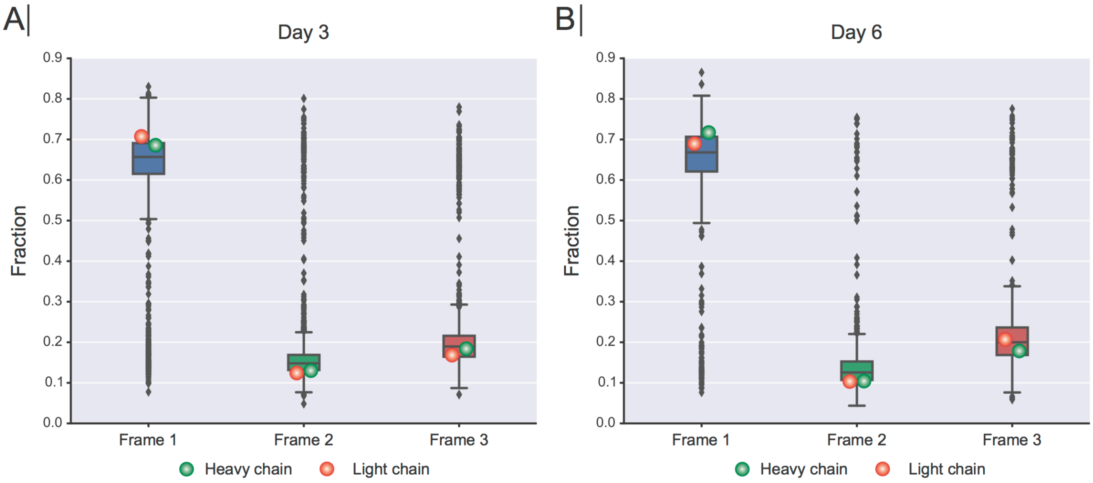

Supplementary Figure S2 Global codon periodicity. To test if the Ribo-Seq data demonstrated a pattern in which pausing occurs on the first base of each codon, we superimposed the ribosomal occupancy for the first, second and third bases in each codon. We included all genes whose protein codon median coverages were greater than 0 in at least one replicate. First, we calculated the percent coverage at each reading frame by summing the coverage at all bases of each frame, divided by total coverage of the whole coding sequence (CDS). Then for each gene, the average frame percentage coverage of biological replicates was used to represent its final frame percentage coverage at day3 and day6.

To substantiate that the codon periodicity of the recombinant mRNAs are equal to that of eEF1a1, we performed an ANOVA analysis. For each gene, ribosomal occupancy, at each base pair of coding region, was normalized as the percentage of total coverage of the codon it belongs to. Following this, an ANOVA test was performed to test if 3 coding frames have the same mean percentages. The ANOVA results show that ribosomal occupancy is significantly different between 3 coding frames.

ANOVA test p-values

	Day3	Day6
heavychain	0	0
lightchain	1.93×10^{-193}	2.62×10^{-170}
Eef1a1	3.41×10^{-322}	0

Figure S3

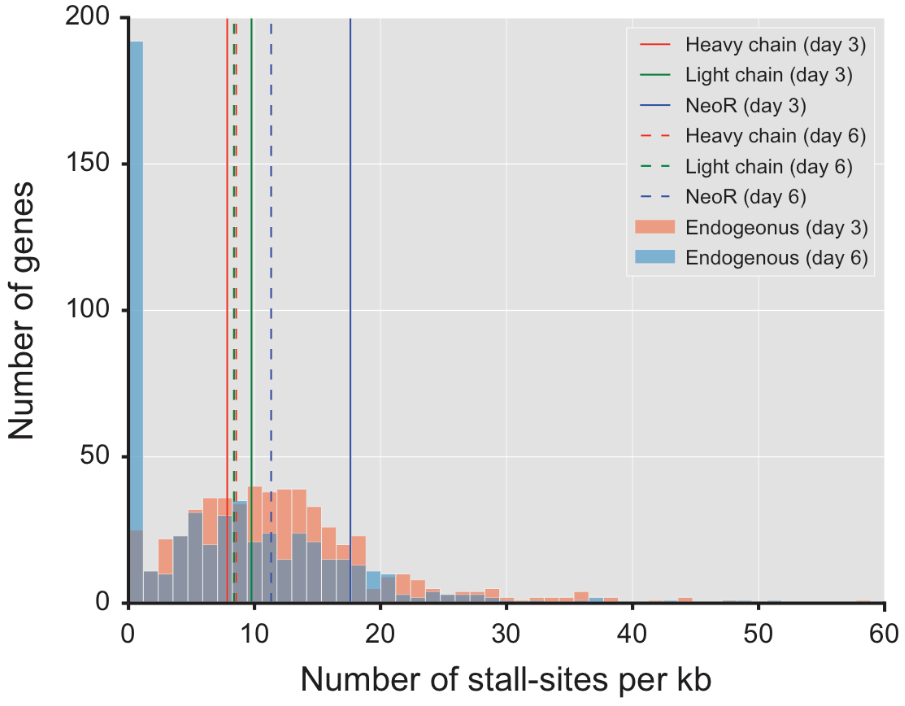

Supplementary Figure S3 Global stalling frequency. To test if the recombinant mRNAs showed increased or decreased ribosomal stalling, we quantified pausing on all genes whose protein codon median coverage were greater than 0 in at least one replicate. For each gene, we calculated the median codon coverage excluding the first 15 and last 10 codons to avoid the bias introduced at those regions. Then coverage at each codon is normalized by the median. The number of stall sites were then counted for each gene, with a normalized density of greater than 25 being considered as a stall sites.

*Note: A recent study has shown that CHX can bias the translation rate of codons [1]. But here we are looking at the overall stall sites instead of codon specificity, therefore the stall sites is comparable.

Figure S4

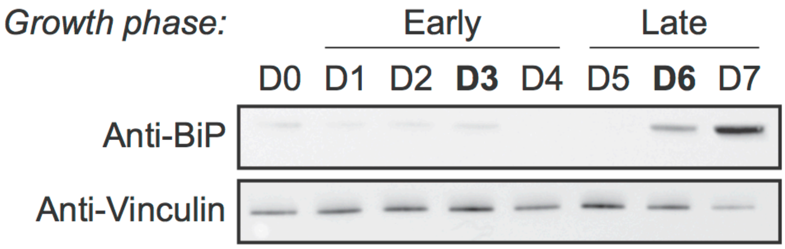

Supplementary Figure S4. Increased BiP expression at late growth phase. Western blot of BiP across the batch culture. Vinculin presented as loading control.

Comment to Figure 4

The NeoR gene was utilized as an initial selection marker before switching to the DHFR selection system to drive up gene copy numbers[2]. Once the transfected cells are exposed to MTX, the cell will be forced to amplify the DHFR cassette to survive the MTX treatment. Because NeoR was encoded from both expression vectors, used to generate the CS13-1.0 cells line, it was most likely co-amplified together with the heavy- and light chain genes. Since NeoR sequesters a substantial amount of translational capacity, it is interesting that the DHFR mRNA only sequestered 0.0002% and 0.0001% of the RNA-Seq reads on day three and day six, respectively (Table S6). This is probably due to the fact that the DHFR gene was is under control of a crippled promoter instead of a strong fully functional promoter as the NeoR gene[2,3]. Since the cells were not grown in the presence of MTX in this experiment, transcription of the crippled DHFR gene would probably decrease significantly. This could explain the low mRNA levels of DHFR observed in our study.

Supplementary statistical analysis to Fig. 3

To our claim that the HC and LC have significantly more reads in the 5'UTR, ribosome occupancy at each base pair of 5'UTR was normalized by the median coverage of coding region, excluding first 45 and last 30 nucleotides. Base pairs with no coverage were excluded from downstream analysis. The Mann-Whitney test was performed to compare coverage at 5'UTR region between recombinant genes and endogenous genes, based on the assumption they have similar median coverage. The results show recombinant median coverages are significantly larger than eef1a1.

	Day3	Day6
Heavychain vs. Eef1a1	1.88×10^{-17}	4.34×10^{-21}
Lightchain vs. Eef1a1	1.33×10^{-11}	6.06×10^{-17}

Supplementary table S1 - Oligos and siRNAs	#	Sequence (5'-3')
**qPCR**		
Chimeric antibody against S surface antigen of hepatitis B virus Heavy Chain_fwd	PR0880	AACAATCTGGACCCGAGCTG
Chimeric antibody against S surface antigen of hepatitis B virus Heavy Chain_rev	PR0881	GCTCTGCTTCACCCACTGAA
Chimeric antibody against S surface antigen of hepatitis B virus Light Chain_fwd	PR0876	AGTGGGTCTGGGACAGACTT
Chimeric antibody against S surface antigen of hepatitis B virus Light Chain_rev	PR0877	CCGAACGTGTACGGAACCTC
NeoR_fwd	PR0964	CTTGCTCCTGCCGAGAAAGT
NeoR_rev	PR0965	CGATGTTTCGCTTGGTGGTC
GAPDH_fwd	PR0580	TTGTCATCAACGGGAAGG
GAPDH_rev	PR0581	GTGAAGACGCCAGTAGATT
**CDS cloning oligos**		
Upsteam CDS (adjacent to CMV TSS)_fwd	PR0864	CTGCTTAACTGGCTTATCGAAA
Downstream CDS (adjacent to BgH poly(A) signal)_rev	PR0907	AGGAAAGGACAGTGGGAGTG
**siRNA***		
NeoR_1_sense		rGrCrGrArArArCrArUrCrGrCrArUrCrGrArGrCrGrArGrCAC
NeoR_1_anti-sense		rGrUrGrCrUrCrGrCrUrCrGrArUrGrCrGrArUrGrUrUrUrCrGrCrUrU

*siRNA designed at:
https://eu.idtdna.com/Scitools/Applications/RNAi/RNAi.aspx

Supplementary table S2 – Heavy- and light chain mRNA sequence*

Light chain:

ACATTGATTATTGACTAGTTATTAATAGTAATCAATTACGGGGTCATTAGTTCATAGCCCATATATGGA
GTTCCGCGTTACATAACTTACGGTAAATGGCCCGCCTGGCTGACCGCCCAACGACCCCCGCCCATTGAC
GTCAATAATGACGTATGTTCCCATAGTAACGCCAATAGGGACTTTCCATTGACGTCAATGGGTGGACTA
TTTACGGTAAACTGCCCACTTGGCAGTACATCAAGTGTATCATATGCCAAGTACGCCCCCTATTGACGT
CAATGACGGTAAATGGCCCGCCTGGCATTATGCCCAGTACATGACCTTATGGGACTTTCCTACTTGGCA
GTACATCTACGTATTAGTCATCGCTATTACCATGGTGATGCGGTTTTGGCAGTACATCAATGGGCGTGG
ATAGCGGTTTGACTCACGGGGATTTCCAAGTCTCCACCCCATTGACGTCAATGGGAGTTTGTTTTGGCA
CCAAAATCAACGGGACTTTCCAAAATGTCGTAACAACTCCGCCCCATTGACGCAAATGGGCGGTAGGCG
TGTACGGTGGGAGGTCTATATAAGCAGAGCTCTCTGGCTAACTAGAGAACCCACTGCTTAACTGGCTTA
TCGAAATTAATACGACTCACTATAGGGAGACCCAAGCTTCATCAGACAGGCAGGGGAAGCAAG**ATGGAT**
TCACAGGCCCAGGTTCTTATGTTACTGCTGCTATGGGTATCTGGTACCTGTGGGGACATTGTGCTGACC
CAATCTCCAGCTTCTTTGGCTGTGTCTCTAGGGCAGAGGGCCACCATCTCCTGCAGAGCCAGCGAAAGT
GTTGATAATTATGGCATTAGTTTTATGAACTGGTTCCAACAGAAACCAGGACAGCCACCCAAACTCCTC
ATCTATACTGCATCCAACCAAGGATCCGGGGTCCCTGCCAGGTTTAGTGGCAGTGGGTCTGGGACAGAC
TTCAGCCTCAACATCCATCCTATGGAGGTGGATGATACTGCAATGTATTTCTGTCAGCAAACTAAGGAG
GTTCCGTACACGTTCGGAGGGGGGACCAAGCTGGAAATAAAACGGACTGTGGCTGCACCATCTGTCTTC
ATCTTCCCGCCATCTGATGAGCAGTTGAAATCTGGAACTGCCTCTGTTGTGTGCCTGCTGAATAACTTC
TATCCCAGAGAGGCCAAAGTACAGTGGAAGGTGGATAACGCCCTCCAATCGGGTAACTCCCAGGAGAGT
GTCACAGAGCAGGACAGCAAGGACAGCACCTACAGCCTCAGCAGCACCCTGACGCTGAGCAAAGCAGAC
TACGAGAAACACAAAGTCTACGCCTGCGAAGTCACCCATCAGGGCCTGAGCTCGCCCGTCACAAAGAGC
TTCAACAGGGGAGAGTGTTAGAGGGAGAAGTGCCCCCACCTGCTCCTCAGTTCCAGCCTGACCCCCTCC
CATCCTTTGGCCTCTGACCCTTTTTCCACAGGGGACCTACCCCTATTGCGGTCCTCCAGCTCATCTTTC
ACCTCACCCCCCTCCTCCTCCTTGGCTTTAATTATGCTAATGTTGGAGGAGAATGAATAAATAAAGTGA
ATCTTTGCACCTGTGGTTTCTCTCTTTCGTCGACCTCGAGGGGGGGCCCTATTCTATAGTGTCACCTAA
ATGCTAGAGCTCGCTGATCAGCCTCGACTGTGCCTTCTAGTTGCCAGCCATCTGTTGTTTGCCCCTCCC
CCGTGCCTTCCTTGACCCTGGAAGGTGCCACTCCCACTGTCCTTTCCTAATAAAATGAGGAAATTGCAT
CGCATTGTCTGAGTAGGTGTCATTCTATTCTGGGGGGTGGGGTGGGGCAGGACAGCAAGGGGGAGGATT
GGGAAGACAATAGCAGGCATGCTGGGGATGCGGTGGGCTCTATGG

* Heavy- and light chain from chimeric antibody against S surface antigen of hepatitis B virus. Bold lettering highlights ORF

Heavy chain:

ACATTGATTATTGACTAGTTATTAATAGTAATCAATTACGGGGTCATTAGTTCATAGCCCATATATGGA
GTTCCGCGTTACATAACTTACGGTAAATGGCCCGCCTGGCTGACCGCCCAACGACCCCCGCCCATTGAC
GTCAATAATGACGTATGTTCCCATAGTAACGCCAATAGGGACTTTCCATTGACGTCAATGGGTGGACTA
TTTACGGTAAACTGCCCACTTGGCAGTACATCAAGTGTATCATATGCCAAGTACGCCCCCTATTGACGT
CAATGACGGTAAATGGCCCGCCTGGCATTATGCCCAGTACATGACCTTATGGGACTTTCCTACTTGGCA
GTACATCTACGTATTAGTCATCGCTATTACCATGGTGATGCGGTTTTGGCAGTACATCAATGGGCGTGG
ATAGCGGTTTGACTCACGGGGATTTCCAAGTCTCCACCCCATTGACGTCAATGGGAGTTTGTTTTGGCA
CCAAAATCAACGGGACTTTCCAAAATGTCGTAACAACTCCGCCCCATTGACGCAAATGGGCGGTAGGCG
TGTACGGTGGGAGGTCTATATAAGCAGAGCTCTCTGGCTAACTAGAGAACCCACTGCTTAACTGGCTTA
TCGAAATTAATACGACTCACTATAGGGAGACCCAAGCTTGGTACCGAGCTCGGATCCACTAGTAACGGC
CGCCAGTGTGCTGGAATTCTGCAGATATCCATCACACTGGCGGCCGCTCTAGAACTAGTGGATCCCCCG
GGCTGCAGGAATTCCGCTGGACTCACAAGTTTTTCTCTTCAGTGACAGACACAGACACAGAACATTCAC
G**ATGTACTTGGGACTGAACTATGTATTCATAGTTTTTCTCTTAAATGGTGTCCAGAGTGAGGTTCAGCT**
GCAACAATCTGGACCTGAGCTGGTGAAACCTGGGGCCTCAGTGAAGATATCCTGCAAGGCTTCTGGATA
TACATTCACTGACTACAACATTCAGTGGGTGAAGCAGAGCCATGGAAAGAGCCTTGAGTGGATTGGATA
TATTTATCCTTACACTGGTGGTACTGGCTACAGCCAGAAGTTCAAGAGCAAGGCCACATTGACTGTAGA
CAATTTCTCCAGCACAGCCTATATGGAACTCCGCAGCCTGACATCTGAGGACTCTGCAGTCTATTACTG
TGCAAGAAACTATGGTTACGACGAGTCTGCTTACTGGGGCCAAGGGACTCTGGTCACTGTCTCTGCAGC
CTCCACCAAGGGCCCATCGGTCTTCCCCCTGGCACCCTCCTCCAAGAGCACCTCTGGGGGCACAGCGGC
CCTGGGCTGCCTGGTCAAGGACTACTTCCCCGAACCGGTGACGGTGTCGTGGAACTCAGGCGCCCTGAC
CAGCGGCGTGCACACCTTCCCGGCTGTCCTACAGTCCTCAGGACTCTACTCCCTCAGCAGCGTGGTGAC
CGTGCCCTCCAGCAGCTTGGGCACCCAGACCTACATCTGCAACGTGAATCACAAGCCCAGCAACACCAA
GGTGGACAAGAAAGTTGAGCCCAAATCTTGTGACAAAACTCACACATGCCCACCGTGCCCAGCACCTGA
ACTCCTGGGGGGACCGTCAGTCTTCCTCTTCCCCCCAAAACCCAAGGACACCCTCATGATCTCCCGGAC
CCCTGAGGTCACATGCGTGGTGGTGGACGTGAGCCACGAAGACCCTGAGGTCAAGTTCAACTGGTACGT
GGACGGCGTGGAGGTGCATAATGCCAAGACAAAGCCGCGGGAGGAGCAGTACAACAGCACGTACCGTGT
GGTCAGCGTCCTCACCGTCCTGCACCAGGACTGGCTGAATGGCAAGGAGTACAAGTGCAAAGGTCTCCA
ACAAAGCCCTCCCAGCCCCCATCGAGAAAACCATCTCAAAGCCAAAGGGCAGCCCCGAGAACCACAGGT
GTACACCCTGCCCCCATCCCGGGATGAGCTGACCAAGAACCAGGTCAGCCTGACCTGCCTGGTCAAAGG
CTTCTATCCCAGCGACATCGCCGTGGAGTGGGAGAGCAATGGGCAGCCGGAGAACAACTACAAGACCAC
GCCTCCCGTGCTGGACTCCGACGGCTCCTTCTTCCTCTACAGCAAGCTCACCGTGGACAAGAGCAGGTG
GCAGCAGGGGAACGTCTTCTCATGCTCCGTGATGCATGAGGCTCTGCACAACCACTACACGCAGAAGAG
CCTCTCCCTGTCTCCGGGTAAATGAGTGCGACGGCCGGCAAGCCCCCGCTCCCCGGGCTCTCGCGGTCG
CACGAGGATGCTTGGCACGTACCCCCTGTACATACTTCCCGGGCGCCCAGCATGGAAATAAAGCACCCA
GCGCTGCCCTGGGCCCCTGCGAGACTGTGATGGTTCTTTCCACGGGTCAGGTCGCTAGAGGGCCCTATT
CTATAGTGTCACCTAAATGCTAGAGCTCGCTGATCAGCCTCGACTGTGCCTTCTAGTTGCCAGCCATCT
GTTGTTTGCCCCTCCCCCGTGCCTTCCTTGACCCTGGAAGGTGCCACTCCCACTGTCCTTTCCTAATAA
AATGAGGAAATTGCATCGCATTGTCTGAGTAGGTGTCATTCTATTCTGGGGGGTGGGGTGGGGCAGGAC
AGCAAGGGGGAGGATTGGGAAGACAATAGCAGGCATGCTGGGGATGCGGTGGGCTCTATGG

Supplementary table S3 - Ribosome profiling alignment offset

Length	Offset
22	7
23	8
24	9
25	10
26	11
27	12
28	15
29	15
30	15
31	15
32	15
33	16
34	16
35	16
36	16
37	16

Reference

1. Hussmann, J. A., Patchett, S., Johnson, A., Sawyer, S. & Press, W. H. Understanding Biases in Ribosome Profiling Experiments Reveals Signatures of Translation Dynamics in Yeast. *PLoS Genet.* **11,** 1–25 (2015).
2. Kim, S. J., Kim, N. S., Ryu, C. J., Hong, H. J. & Lee, G. M. Characterization of chimeric antibody producing CHO cells in the course of dihydrofolate reductase-mediated gene amplification and their stability in the absence of selective pressure. *Biotechnol. Bioeng.* **58,** 73–84 (1998).
3. Ryu, C. J. *et al.* In Vitro Neutralization of Hepatitis B Virus by Monoclonal Antibodies Against the Viral Surface Antigen. **233,** 226–233 (1997).
